# Plant defense elicitors: plant fitness versus wheat stem sawfly

**DOI:** 10.7717/peerj.5892

**Published:** 2018-11-01

**Authors:** Govinda Shrestha, Shabeg S. Briar, Gadi V.P. Reddy

**Affiliations:** Department of Research Centers, Western Triangle Agricultural Research Center, Montana State University, Conrad, MT, USA

**Keywords:** Actigard, Insect fitness, Plant fitness, Mortality

## Abstract

The wheat stem sawfly (WSS), *Cephus cinctus* Norton, is an important wheat pest in the Northern Great Plains of the USA. No single control measure effectively suppresses WSS damage. This study provides information on the effects on the WSS adult settling preference behavior on wheat plants under laboratory conditions from treatment with both synthetic plant defense elicitors (Actigard^®^ and *cis*-jasmone) and a botanical insecticide (Azadirachtin^®^). In addition, field experiments were performed to determine whether these chemicals impact the WSS fitness (larval mortality and larval body weight), winter wheat plant fitness (infestation, stem lodging, yield, and quality), adult population of WSS and *Bracon* spp., and larval parasitism levels. Our lab results showed that there were no significant differences in adult settling behavior on plants exposed separately to each chemical and control. In contrast, when adults were exposed simultaneously to treated and untreated plants, there was a significant reduction in the percentage of adults settling on Actigard^®^ and Azadirachtin^®^ treated plants compared to plants sprayed with water in the same cage. However, in field situations, regardless of application timing and field location, none of the chemicals significantly reduced adult population or stems damage. The exception was two times applications of Actigard^®^ had significantly lower WSS infested stem damage levels at 30 days after initial treatment applications at Knees and 50 days at Choteau locations compared to control, but without effect at the Conrad location. The field study indicated that two times applications of Actigard^®^ significantly increased diapausing larval mortality percentages and lowered stem lodging levels compared to untreated controls at Knees and Choteau locations, while no effects at Conrad location. Larval body weight was significantly lower in plots treated with Actigard^®^ at Knees and Conrad, but no effects at Choteau. No significant differences were found in wheat yield and quality in plots treated with chemicals and controls at any location. *Bracon* spp. adult population and parasitism levels were not negatively affected by the use of chemicals. In conclusion, this study offers insights on what treatments should be emphasized in more detail despite variable findings.

## Introduction

Wheat (*Triticum aestivum* L.) is the most widely grown cereal crop world-wide, and U.S. yields amount to 47 million tons annually (USDA NASS, 2017). In the Northern Great Plains of the USA (Montana, North Dakota, South Dakota, Colorado, Wyoming, and Nebraska), 6.48 million hectares were planted to wheat in 2017. Wheat is an important source of protein and calories, supplying nearly 20% of daily protein and food calories globally (Wheat Initiative, 2015).

One of the most important insect pests of wheat in the Northern Great Plains of the USA and Canada is the wheat stem sawfly (WSS), *Cephus cinctus* Norton (Hymenoptera: Cephidae) (Varella et al., 2015; Portman et al., 2018). *Cephus cinctus* larvae feed inside wheat stems, causing significant reductions in plant photosynthesis (Macedo et al., 2005), kernel weight (Morrill et al., 1994), grain yield, and grain quality (Morrill et al., 1994). Grain yield losses are also increased by plant lodging, when mature larvae cut grooves around the inside perimeter of the lower part of wheat stems, making harvesting operations difficult (Criddle, 1922). Annual economic loss caused by *Cephus cinctus* damage to wheat is estimated to be $100–350 million in the USA and Canada (Beres et al., 2011). In addition, WSS also damages barley (*Hordeum vulgare* L.) and rye (*Secale cereal* L.) (Wallace & McNeal, 1966; Beres et al., 2011; Varella et al., 2018).

The principal pest management approaches to reduce WSS damage are planting solid-stemmed wheat varieties and some cultural practices (Beres et al., 2011). However, resistant wheat varieties have not been widely adopted by Montana wheat growers, likely because of their lower yield and protein levels, and variable performance under different environmental conditions (Berzonsky et al., 2010; Szczepaniec, Glover & Berzonsky, 2015). Cultural practices such as tillage (in spring or fall) sometimes reduces WSS populations (Beres et al., 2011), but requires additional field operations, which increases production costs (Shanower & Hoelmer, 2004). An organophosphate insecticide (Thimet 20-G^®^) has recently been registered in Montana against *Cephus cinctus* (Wanner & Tharp, 2015), but this chemical poses many health and environmental risks (Lerro et al., 2015).

Two idiobiont larval parasitoids, *Bracon cephi* (Gahan) and *Bracon lissogaster* (Muesebeck) (Hymenoptera: Braconidae) are important natural enemies of *Cephus cinctus*. These two parasitoids can parasitize up to 98% of WSS larvae, but are most effective when WSS populations are low (Morrill, Kushnak & Gabor, 1998; Shanower & Hoelmer, 2004). Larvae and adults of the Clerid beetle *Phyllobaenus dubius* (Wolcott) (Coleoptera: Cleridae) are predators of WSS larvae, but their potential for controlling the pest in the field is undetermined (Morrill et al., 2001). The only commercially available biopesticide tested for WSS management is the entomopathogenic nematode *Steinernema feltiae* (Nematoda: Rhabditida), mixed with an adjuvant (Penterra), which was able to penetrate wheat stubble and kill diapausing larvae under field conditions in Montana (Portman, Krishnankutty & Reddy, 2016). While biological control is a promising strategy for controlling WSS populations in some regions (Tangtrakulwanich et al., 2014; Portman et al., 2018), additional measures are needed. The development of synthetic plant defense elicitors and botanical insecticides such as Azadirachtin^®^ may have potential to enhance control of this significant wheat pest in the Northern Great Plains of the USA and Canada. In addition, they may also serve as sustainable alternatives to costly and harmful synthetic chemical insecticides (Isman, 2006; Bektas & Eulgem, 2015).

Synthetic plant defense elicitors are small molecules that can induce defense responses in plants like that activated by natural herbivory or pathogen infection (Karban & Kuc, 1999). Jasmonic acid (JA) and salicylic acid (SA) are two of the most important and common plant hormones that induce resistance in plants against herbivores and pathogens, respectively. JA is derived from linolenic acid via the octodecanoid pathway. Plants emit JA when they are attacked by herbivores and this release triggers an increase in proteins and secondary metabolites that provide resistance against insect pests. However, JA production in plants is often low, and thereby failing to counteract pest attack (Creelman & Mullet, 1995; León, Rojo & Sánchez-Serrano, 2001). Exogenous application of JA or its methyl ester (methyl jasmonate) have shown to induce resistance to several insect pests in various crops, for example, resistance to green peach aphid *Myzus persicae* (Sulzer) on tomato (Cooper, Jia & Goggin, 2004), cotton aphid *Aphis gossypii* (Glover) on cotton, (Omer et al., 2000), and brown plant hopper *Nilaparvata lugens* (Stal) on rice (Senthil-Nathan et al., 2009). JA application in wheat reduced wheat midge, *Sitodiplosis mosellana* (Gehin) populations (El-Wakeil, Volkmar & Sallam, 2010; Shrestha & Reddy, 2017). Application of methyl jasmonate in wheat lowered the feeding preferences of both bird cherry-oat aphid, *Rhopalosiphum padi* L. (Slesak, Slesak & Gabrys, 2001) and grain aphid, *Sitobion avenae* (Fabricius) (Cao et al., 2014). *cis*-jasmone is another well-known component of plant volatiles, and it is released naturally from insect-damaged plants (Birkett et al., 2000). It is structurally related to JA and methyl jasmonate and known to activate plant defense. Laboratory and field experiments have shown that exogenous application of *cis*-jasmone to plant surfaces induce resistance against many insect pests such as beet armyworm *Spodoptera exigua* (Hübner) (Disi et al., 2017), western flower thrips *Frankliniella occidentalis* (Pergande) (Egger & Koschier, 2014) and *S. avenae* (Bruce, Pickett & Smart, 2003). *cis*-jasmone induction resulted in wheat plants becoming less favorable for *Sitobion avenae* by reducing aphid settlement, growth and development (Bruce, Pickett & Smart, 2003).

Salicylic acid is also a critical plant hormone that induces resistance in plants to fungal, bacterial, and viral pathogens. The plant elicitor benzo-(1,2,3)-thiadiazole-7-carbothioic acid *S*-methyl ester (BTH), commercialized with product name–BION^®^ (in Europe) and Actigard^®^ (in the USA), is a functional analog of SA. This synthetic plant elicitor has been known to induce systematic acquired resistance in a variety of plants including monocots (e.g., wheat, maize) and dicots (e.g., tobacco, tomato, pepper) (Vallad & Goodman, 2004). This product was primarily developed for disease control in a variety of agricultural crops, conferring resistance to a broad range of fungal, bacterial and viral pathogens. However, BTH foliar applications have also been shown to confer plant resistance to insect herbivores such as, aphids (Cooper, Jia & Goggin, 2004), whiteflies (Correa et al., 2005), and leaf miners (Moran & Thompson, 2001).

Several botanical pesticides are toxic and/or repellent to agricultural insect pests, especially plant extracts from plant species of Asteraceae, Rutaceae, and Meliaceae (Isman, 2006). Azadirachtin^®^ is one of the major botanical pesticides that contains a complex tetranortriterpenoid limonoid as an active ingredient and extracted from neem (*Azadirachta indica* A. Juss) tree seeds. It displays a range of effects on insect pests, including (1) insect behavior, acting as oviposition deterrent, repellent and antifeedant; (2) insect physiology, reducing insect growth and reproduction, delaying developmental period and increasing mortality. Presently, Azadirachtin^®^ is a prominent natural pesticide and represents an alternative to synthetic pesticide. It has been explored for its potential to improve management of a variety of agricultural crop insect pests, such as diamondback moth, *Plutella xylostella* L. (Liang, Chen & Liu, 2003), cotton leafworm, *Spodoptera littoralis* Boisduval (Martinez & Van Emden, 1999) and WSS (Tangtrakulwanich et al., 2014). In addition to controlling insect pests, Azadirachtin^®^ has minimal impacts on natural beneficial insects and low environmental impact (Mordue & Nisbet, 2000).

In this study, two commercially available synthetic defense plant elicitors (Actigard and *cis*-jasmone) and a botanical insecticide (Azadirachtin^®^) were evaluated for their ability to repel WSS adult settling behavior, and thus can provide protection for wheat from pest oviposition, under laboratory conditions. WSS adult settling behavior refers to whether adults have abilities to distinguish chemical treated and untreated plants and measured through percentage of adults settled (e.g., landing, resting, and walking) on plants. Understanding WSS adult behavior is particularly important to know since a previous study showed that they have the ability to determine a suitable wheat variety for oviposition when they have a choice with unsuitable or a least preferred variety (Weaver et al., 2009). The effects of these chemicals on WSS fitness (larval mortality and larval body weight) and winter wheat plant fitness (infestation, stem lodging, yield, and quality) was assessed under field conditions. This study is a first step in identifying synthetic defense plant elicitors and Azadirachtin^®^ for WSS management. In addition, the impact of these chemicals on *Bracon* species adult population levels and larval parasitism percentages was examined.

## Materials and Methods

### Insects

Wheat stubble containing diapausing WSS larvae were collected from winter wheat fields in Pondera and Chouteau counties, Montana, from December 2016 to February 2017. Infested WSS stems (8–10 cm long) were stored in 473 ml round plastic deli containers and maintained in a climate cabinet (5 ± 1 °C) for up to 3 months in the dark to facilitate the completion of obligatory larval diapause (Holmes, 1977). Afterward, WSS-infested stems were transferred to new deli containers that had been half filled with garden soil, with the stem inserted upright into the soil. A total of 50–60 stems were placed in each container, kept inside insect cages (12 × 10 × 10 cm) and held at 19–21 °C, 50–60% RH and a 16:8 h L:D photoperiod at the Western Triangle Agricultural Research Center (WTARC) of Montana State University, USA.

Containers were lightly moistened twice a week with tap water to minimize desiccation of the WSS larvae. Cages were checked daily, and newly emerged WSS adults were transferred into new empty cages with pieces of cotton moistened with a 10% of honey water solution until they were used for experiments. Approximately 100 WSS adults were held in each cage, with a 60:40 female:male ratio. To minimize the effect of host deprivation time, laboratory bioassays were performed with WSS females within 48 h of emergence. In this rearing system, adults emerged within 3–4 weeks from the WSS infested stems.

### Plants

All experiments (field and laboratory) used the winter wheat variety “Yellowstone” (Bruckner et al., 2007), because this is one of the most common wheat varieties grown in Montana. This variety is high yielding, has excellent baking and noodle qualities, moderate resistance to plant diseases (dwarf smut and stripe rust), and is highly susceptible to WSS damage (USDA NASS, 2017).

The winter wheat seedling nursery plot was established at WTARC before the laboratory bioassay experiments because wheat plants require exposure to cool temperatures for stem elongation and reproduction. The consideration for establishing a nursery plot was mainly because of low winter wheat seedling survival rate when a refrigerator was used to complete the vernalization process (G. Shrestha, personal observation, 2016). The plot was seeded at a rate of 194 live seeds per m^2^ in September 2016. Nutrient and irrigation management followed standard local growing practices. When the plants completed the vernalization process in spring 2017, the seedlings (two unfolded leaves) were transplanted into tapered square pots (13 × 13 × 13.5 cm) at a density of three plants per pot. Plants were maintained in a greenhouse at 18–20 °C, 50–60% RH and natural light conditions until used for experiments. Each pot contained 1.62 kg of prepared soil mixture, which was an equal proportion of sand, vermiculite, and peat moss, and N, P and K level of 17.13, 11.49, and 8.10 g/100 kg of soil mixture, respectively. Plants were watered four to five times weekly, and fertigated with Peters General Purpose Fertilizer (JR Peters, Allentown, PA, USA) at 100 ppm in aqueous solution at fortnight intervals. Nutrient application started when plants reached the third leaf stage (Zadoks, Chang & Konzak, 1974). Plants used for the laboratory experiments were at developmental stage “Zadoks 33” at which two to three nodes are visible, since WSS female adults prefer this stage for oviposition (Holmes & Peterson, 1960).

### Chemical source and rate

Actigard 50WG^®^ was obtained from Syngenta (Fargo, ND, USA) as a water-dispersible granular formulation containing 50% active ingredient. *cis*-jasmone was obtained from Sigma-Aldrich (St. Louis, MO, USA) as a soluble liquid formulation with 85% purity. Azadirachtin^®^ (extracts from neem), as a 1.2% emulsifiable concentration, was obtained from Gowan Company (Yuma, AZ, USA). For both laboratory and field experiments, the chemicals were applied at concentrations of 0.75 ml/l (Actigard), 0.5 ml/l (*cis*-jasmone) and 2.88 ml/l (Azadirachtin), the concentrations recommended by manufacturers for agricultural use.

### Laboratory experiments: sawfly adult settling preference

Bioassay experiments were performed to determine the settling preference of WSS female adults between wheat plants treated with chemicals (as above) and an untreated control. For the application of chemicals, potted plants established in the greenhouse were transported to a spraying room. Spray application was made to individual plants using a 750 ml hand-held sprayer, with a spray volume of 20 ml per plant. After chemical application, plants were allowed to dry for 1 h, and then placed in collapsible cages. Control plants were treated with tap water using the same device.

Four wheat plants were placed inside each cage. Two plants were those that had been treated with one of the test chemicals, while two were controls treated with water. A total of 10 female adults (48 h old) were released in the center of each cage and allowed to settle overnight. However, insects were released 5 days after the Actigard treatment application to allow plants to induce resistance against insect pests (Benhamou & Bélanger, 1998). The following day, the position of each WSS adult (on chemically treated plants, water treated plants, or elsewhere) inside the test cage was recorded, once in the morning (9:00–10:00 am) and once in the afternoon (15:00–16:00 pm).

A similar procedure was followed to assess the settling behavior of WSS female adults under a no-choice condition, in which a single plant treated with one of the chemicals or with water (control) was placed in a cage and seven insects were released. The caged plants were held at 19–21 °C, 50–60% RH, and a 16:8 h L:D photoperiod. Each treatment was replicated four times (each insect cage = one replicate) per experiment, and the experiment was conducted twice (*N* = 8).

### Locations of winter wheat fields used in field trials

The field experiment was conducted at three locations: Knees (N 48°00′08.5 W 111°21′51.8), Conrad (N 48°18′29.0 W 111°55′23.1), and Choteau (N 47°59′36.0 W 112°06′49.9), in the Golden Triangle, Montana, USA. All experimental locations were known to have had moderate to high WSS infestations for many years. The experimental plots were seeded between the first and second week of September 2016 at a rate of 194 live seeds per m^2^. The seeds were planted in four rows, with 30 cm between rows. Glyphosate (Roundup Powermax^®^) was applied at the rate of 2.5 l/ha (the active ingredient of 540 g/l of acid glyphosate) before seeding to control weed growth. Fertilizers N, P, and K at 224.2, 0, and 22.4 kg/ha were broadcasted while planting, and an additional application of 12.3, 25.2, and 0 kg/ha of these three nutrients were applied through the seed plot drill.

At each field location, treatments were arranged in a randomized complete block design with four replicates per treatment. Plots for treatments were 3.6 × 1.2 m separated by 0.60 m buffer zones to avoid cross contamination of treatments.

### Monitoring of wheat stem sawfly adults

WSS has a single generation per year. Adults have mean life expectancy of only 5–8 days (Criddle, 1922), but adult emergence can occur over an extended period of time, generally about 3 weeks. WSS female adults usually begin to deposit eggs inside developing wheat stems when stem elongation starts during the spring season (Knodel, Shanower & Beauzay, 2016; Fulbright et al., 2017). Multiple eggs can be laid within a stem, but only a single larva survives up to maturity. Eggs hatch about 5–7 days after being laid. Larval development includes three to four stages and will last for about 1 month. As wheat plants mature, the larva moves toward the base of the stem to prepare for a period of dormancy called diapause (Fulbright et al., 2017). Further information regarding WSS life cycle can be obtained from Knodel, Shanower & Beauzay (2016) and Fulbright et al. (2017).

Estimating the ideal application time for synthetic plant defense elicitors and a botanical insecticide (Azadirachtin^®^) can be one of the critical factors for WSS management. Currently, no degree-day model has been established for determining adult emergence (Knodel et al., 2009). Therefore, two methods were used for monitoring the emergence of adults: (1) dissection of WSS-infested stubble to determine the stage of immature development (Nansen et al., 2005) and (2) sweep net sampling in the winter wheat fields to detect adults (Knodel et al., 2009). Experimental plots and their adjacent winter wheat fields were scouted weekly from the last week of April until mid-June, 2017.

### Field trial: application of chemicals

**Figure 1:**
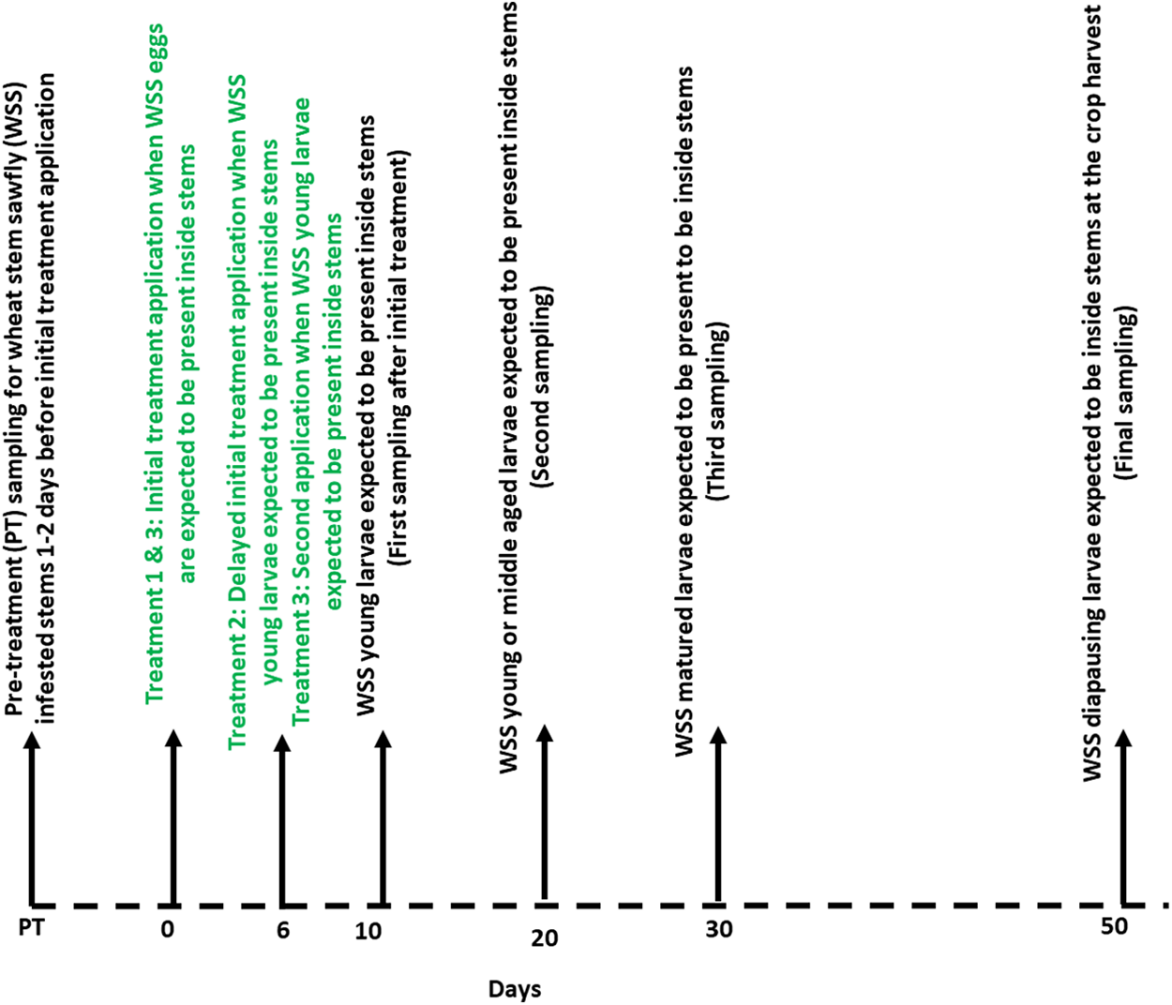
Timeline showing the synthetic plant defense elicitors (Actigard^®^ and *cis*-jasmone) and Azadirachtin^®^ applications and sampling of wheat stem sawfly infested stems relative to expected life stages.

The field experiment included three treatment types: (1) initial application of chemicals when WSS eggs expected to be present inside stems (treatment # 1), (2) delayed initial application of chemicals when WSS young larvae are expected to be present inside stems (treatment # 2) and (3) initial and delayed initial applications of chemicals (i.e., two times) when WSS eggs and larvae are expected to be present, respectively, inside stems (treatment # 3) (Fig. 1). The goal of the treatment categories was to determine the most vulnerable insect stages and the response of wheat plants to the spray timing. All chemicals were applied on the same date (treatment 1: May 29, 2017; and treatment 2 & 3: June 6, 2017) at the Knees and Choteau field trial locations. However, for the Conrad location, spraying started 10 days later because of the late emergence of WSS adults. Treatments were applied using a SOLO backpack sprayer (SOLO, Newport News, VA, USA) calibrated to deliver about 150 l of spray solution/ha based on nozzle flow and walking speed. Plants treated with water served as untreated control plots. At all field trial locations, chemicals were applied at the wheat stage with four to six nodes.

### Collection of wheat stems

Wheat stems were sampled in all plots to measure treatment effects during the growing season. Samples were collected 3 days before treatment application, and 10, 20, 30, and 50 days after initial treatment application. Three random samples were taken from the two central rows of each treatment plot, with five stems/sample. Wheat stems were cut from the base of plants with a scissors, placed into a single zip-lock plastic bag, and transported from the field site in coolers. During the final sampling, clumps of stems were removed randomly at each of three sampling points in the two middle rows of each plot with the help of a shovel to collect whole, mature plants. This technique was used mainly because diapausing WSS larvae prefer to remain at the base of the wheat stem.

Samples were taken to the laboratory, where stems were dissected lengthwise with a fine bladed scalpel to measure (1) WSS infestation levels, including the presence of WSS immatures, or WSS frass inside dissected wheat stems at each sampling time, (2) WSS diapausing larval body weights and their mortality (number of dead larvae inside stems) at the final sampling time, and (3) WSS larval parasitism percentage, as measured by the presence of parasitoid cocoons vs unparasitized WSS stages inside wheat stems.

### Sampling of adult WSS and parasitoids

To determine whether synthetic plant defense elicitors or Azadirachtin repelled WSS adults and what further impacts these compounds might have on WSS parasitoids, a sweep net was used to sample adult insects, both WSS and the two *Bracon* parasitoid species. Sweeping was done with a standard sweep net (180° arc), collecting 15 sweeps from each treatment plot. Netting was done 10, 20, and 30 days after initial treatment application. Collected insects were stored in a freezer until they were examined and counted in the laboratory.

### Stem lodging level at harvest

Wheat stem lodging was determined by visually classifying locations in pots on a rating scale from 1 to 10, with 1 indicating that all plants in a plot were vertical and 10 indicating that all plants in a plot were horizontal during crop harvest time.

### Yield and quality

A Hege 140 plot combine (Hege Maschinen GmbH, Lichtenstein, Germany) was used to harvest the wheat from treatment plots. Care was taken to avoid the borders and any overlap of treatment effects on wheat yield and quality. Each plot length was measured, and the wheat grain threshed from each plot. Wheat grains were cleaned with a seed processor (Almaco, Avenue Nevada, IA, USA) and weighed on a scale to determine yield. Test weight was measured on a Seedburo test weight scale (Seedburo Equipment Co, Des Plaines, IL, USA). The protein percentages of seeds were determined with NIR grain analyzer, IM 9500 (Perten Instruments, Springfield, IL, USA).

### Data analysis

The data were analyzed using R 2.15.1 (R Development Core Team, 2011). For all data, a normal quantile–quantile plot was performed to validate normality of data and equality of variance. Wherever appropriate, Tukey’s contrast pairwise multiple comparison was run using the function “multcomp” to compare significant differences in means (Hothorn, Bretz & Westfall, 2008).

### Adult settling behavior

Data was pooled over the two experimental runs as there was no significant difference (>0.05) between two experimental runs. Two-way analysis of variance (ANOVA) was used to determine the effects of treatment and time on WSS adults settling behavior when adults were exposed separately to each chemical and control inside insect cages. Paired *t*-test was used to assess the effect of treatment on WSS adults settling behavior at morning or evening observation, when adults were exposed simultaneously to each chemical treated and untreated plants inside cages.

### WSS fitness: larval body weight and mortality

The overall data on larval body weight was fitted to a mixed model with chemical type and application time as fixed factors, the location as random effect (1|Unit) and the larval body weight per plot as response variable using the function “lmer.” The model was subsequently reduced if applicable, with stepwise elimination of factors having no effect. Kenward–Roger test was carried out to compare the models with the function “KRmodcomp” (Halekoh & Højsgaard, 2012). In addition, data of WSS larval body weight for each location was fitted to a generalized linear model with the function “glm” and family “gaussian.” Two categorical variables of data, chemical type and application timing were converted into factors. The mean larval body per treatment was the response variable. The model was then modified with stepwise removal of factors having no effect and the *F*-test was performed to compare the models. A similar procedure was followed for analysis of WSS larval mortality data.

### Winter wheat plant fitness: lodging, infestation, yield and quality

Stem lodging data were analyzed with a similar method as described for WSS fitness. One-way ANOVA was carried out to determine effect of treatment on: (1) WSS infested stem damage percentage at each sampling time including pre-treatment data and (2) grain yield and quality.

### Wheat stem sawfly adults, and parasitoid adults and their parasitism level

The numbers of WSS and parasitoid adults, and parasitism (*Bracon* spp. pupae inside stems) percentage data were non-normally distributed even after log transformation, and a non-parametric one-way ANOVA (Kruskal–Wallis test) was used to examine application timing of each treatment on (1) numbers of WSS and parasitoid adults at each sampling time and (2) parasitism percentage at the final sampling time. Mann–Whitney *U*-tests were used as post hoc multiple comparisons between treatment means.

## Results

### Wheat stem sawfly adult settling preferences

**Figure 2:**
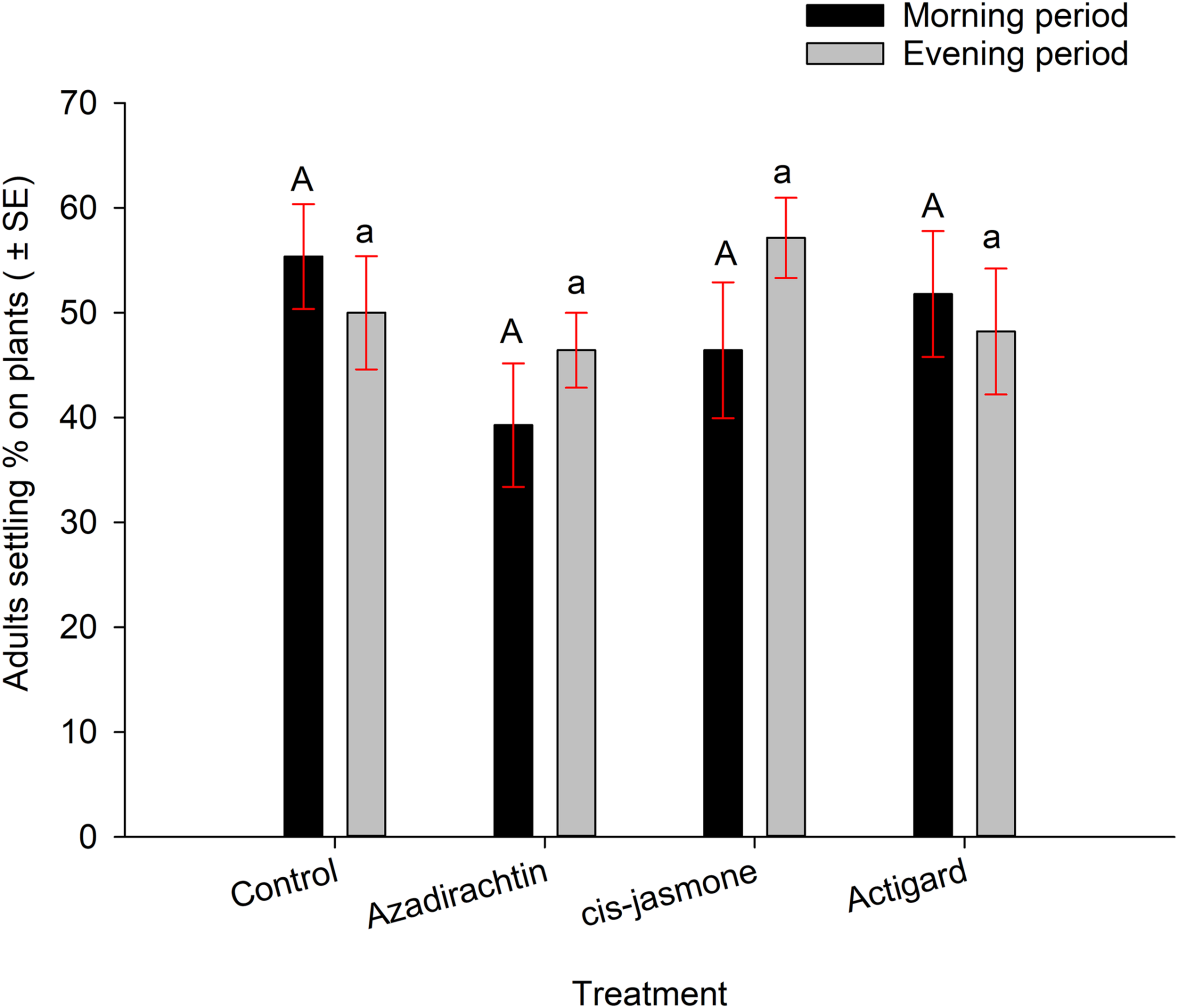
Effects of synthetic plant defense elicitors (Actigard^®^ and *cis*-jasmone) and Azadirachtin^®^ applications on wheat stem sawfly adults settling behavior when adults were exposed separately to each chemical and control inside cages. Bars that share the different upper case (morning period) and lower case (evening period) letters are significantly different (Tukey test, *P* < 0.05). Data pooled over the two experimental runs. The total numbers of replicates were eight per treatment.

Wheat stem sawfly adults were able to settle (preparatory to oviposition) on winter wheat seedlings when plants treated with each chemical and untreated control presented to adults, either separately or together inside insect cages. In no-choice experiments, average adult settling percentages were higher on plants treated with untreated control (53%) followed by *cis*-jasmone (50%), Actigard^®^ (49%) and Azadirachtin^®^ (42%) (Fig. 2). However, there was no significant difference between in adult settling percentage between the treatments (*F* = 1.20; d*f* = 3, 56; *P* = 0.32). The observation time had further no effect on adult settling percentage (*F* = 1.49; d*f* = 1, 56; *P* = 0.22) (Fig. 2). There were no significant interaction effects between treatments and observation time (*F* = 0.57; d*f* = 3, 56; *P* = 0.63).

**Figure 3:**
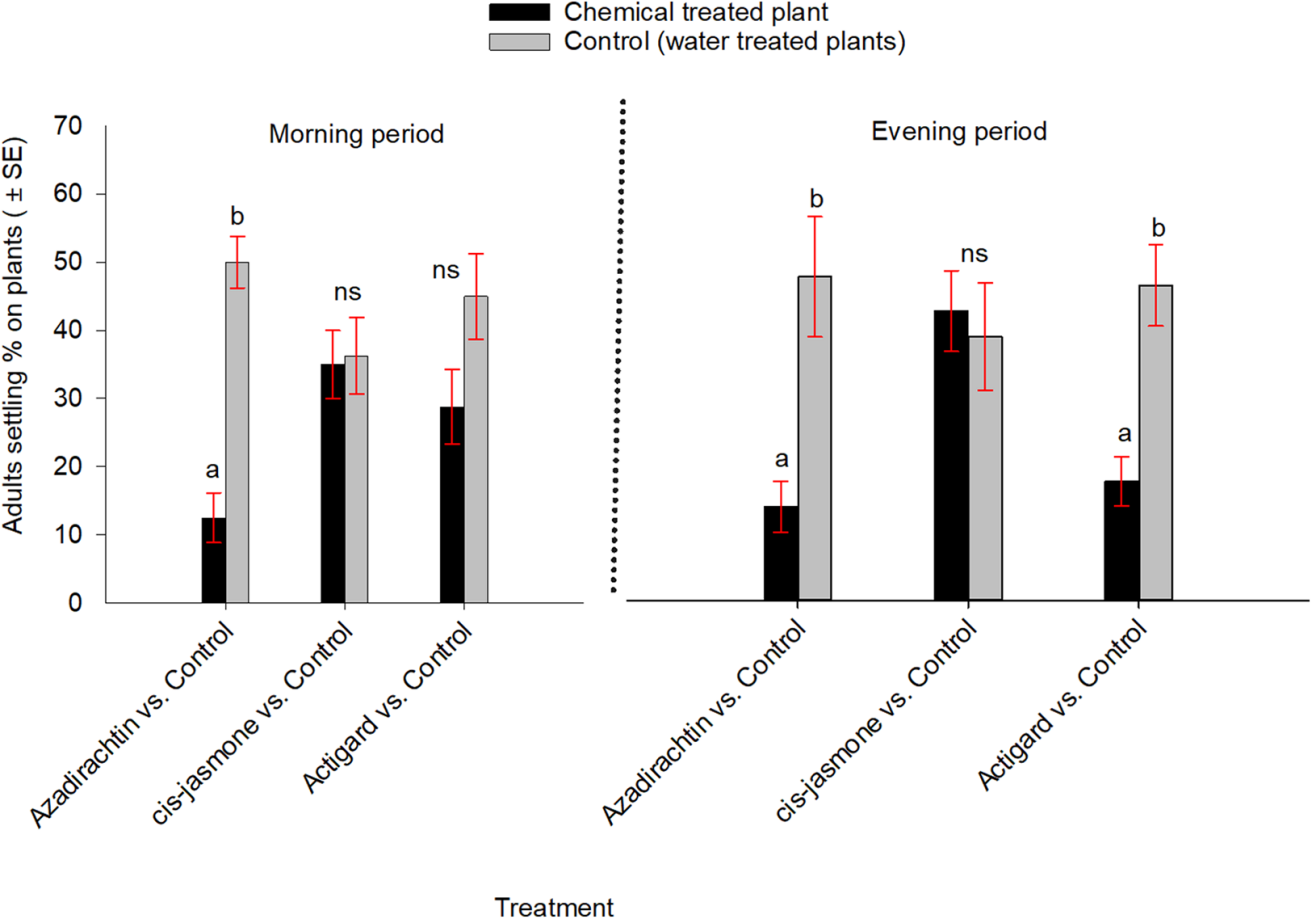
Effects of synthetic plant defense elicitors (Actigard^®^ and *cis*-jasmone) and Azadirachtin^®^ applications on wheat stem sawfly adults settling behavior when adults were exposed simultaneously to treated and untreated plants inside cages under laboratory conditions. Bars bearing different letters within each treatment group comparisons (chemical vs control) at the morning and evening periods are significantly different (Paired *t*-test, *P* < 0.05). Data pooled over the two experimental runs. The total numbers of replicates were eight per treatment group.

In contrast, when WSS adults were exposed simultaneously to each chemical treated and untreated plants inside cages, they significantly preferred untreated control over Azadirachtin-treated plants both in the morning (*t* = −5.79; d*f* = 7; *P* = 0.001) and evening (*t* = −3.00; d*f* = 7; *P* = 0.02) observation periods (Fig. 3). Adults also showed significantly higher preference to the untreated control than the Actigard-treated plants in the evening (*t* = −3.66; d*f* = 7; *P* = 0.01), but without effect in morning (*t* = −1.47; d*f* = 7; *P* = 0.18) observation period (Fig. 3). Adults showed no significant preferences between untreated control and *cis*-jasmone treated plants in either the morning (*t* = −0.11; d*f* = 7; *P* = 0.90) or evening (*t* = 0.28; d*f* = 7; *P* = 0.78) observation periods (Fig. 3).

### Infestation level

Pre-treatment data is presented in Table 1. At the Knees location, WSS infested damage stem percentage levels were generally low in chemical-treated plots compared to untreated control plots, irrespective of sampling time (Table 1). However, there were no significant differences in WSS infested damage percentage levels between treated and untreated control plots for most of the sampling times (10, 20, and 50 days after initial treatment applications) (Table 1). Except for third sampling time (i.e., 30 days after initial treatment application), Actigard^®^ two applications (WSS egg and larval stages) had a significantly lower stem damage percentage levels (mean ± SE; 35.00% ± 9.18) than the untreated control plots (67.00% ± 7.20 to 77.00% ± 1.92) (Table 1).

**Table 1:** Effects of synthetic plant defense elicitors (Actigard^®^ & *cis*-jasmone) & Azadirachtin^®^ applications on wheat stem sawfly (WSS) infested stems % level (mean ± SE) in winter wheat fields at the three study locations of Montana.

Treatment		WSS infested stems percentage			
	PT	10 DAT	20 DAT	30 DAT	50 DAT
**Knees**					
Water-ES	23 ± 5.77	38 ± 12.58	55 ± 6.31	67 ± 7.20ab	74 ± 8.57
Water-ELS	20 ± 8.16	42 ± 5.00	65 ± 7.39	72 ± 4.19ab	73 ± 8.66
Water-LS	23 ± 7.93	40 ± 6.09	52 ± 7.39	77 ± 1.92ab	81 ± 3.88
Actigard-ES	25 ± 4.71	33 ± 7.20	38 ± 9.57	62 ± 3.19ab	79 ± 10.5
Actigard-ELS	22 ± 10.67	27 ± 6.09	35 ± 6.31	35 ± 9.18c	67 ± 5.31
Actigard-LS	13 ± 5.69	25 ± 4.19	58 ± 3.19	53 ± 5.44ab	78 ± 8.95
*cis*-jasmone-ES	27 ± 7.20	35 ± 3.19	58 ± 9.18	70 ± 8.39ab	81 ± 6.47
*cis*-jasmone-ELS	20 ± 5.69	17 ± 4.30	48 ± 10.67	55 ± 5.69ab	80 ± 8.65
*cis*-jasmone-LS	17 ± 7.93	37 ± 4.30	47 ± 9.81	67 ± 7.20ab	72 ± 11.00
Azadirachtin-ES	22 ± 5.69	23 ± 10.36	38 ± 7.39	60 ± 0.00ab	85 ± 1.08
Azadirachtin-ELS	18 ± 3.19	25 ± 5.00	50 ± 6.38	55 ± 7.39ab	63 ± 12.77
Azadirachtin-LS	17 ± 7.93	33 ± 8.16	48 ± 8.76	63 ± 10.00ab	77 ± 9.76
**Statistical output**	*F*_11,36_ = 0.33; *P* = 0.97	*F*_11,36_ = 1.25; *P* = 0.29	*F*_11,36_ = 1.31; *P* = 0.26	*F*_11,36_ = 2.88; *P* = 0.001	*F*_11,36_ = 0.51; *P* = 0.89
**Choteau**					
Water-ES	17 ± 10.36	57 ± 12.32	67 ± 4.17	77 ± 4.30	93 ± 2.25a
Water-ELS	25 ± 3.19	48 ± 11.01	62 ± 11.67	82 ± 8.77	96 ± 1.65a
Water-LS	33 ± 5.44	58 ± 3.19	67 ± 6.09	87 ± 2.72	92 ± 4.52a
Actigard-ES	20 ± 6.94	38 ± 7.39	55 ± 3.19	72 ± 7.88	96 ± 2.87a
Actigard-ELS	18 ± 7.39	33 ± 4.71	58 ± 8.33	85 ± 5.69	80 ± 1.5b
Actigard-LS	18 ± 5.00	35 ± 5.69	53 ± 8.16	68 ± 11.98	83 ± 4.86a
*cis*-jasmone-ES	28 ± 7.39	53 ± 6.09	53 ± 2.72	72 ± 6.87	89 ± 5.12a
*cis*-jasmone-ELS	18 ± 10.32	23 ± 8.82	77 ± 8.39	82 ± 11.34	90 ± 4.59a
*cis*-jasmone-LS	17 ± 8.82	63 ± 12.91	70 ± 1.92	85 ± 8.77	97 ± 1.32a
Azadirachtin-ES	22 ± 5.00	48 ± 8.33	60 ± 9.81	78 ± 5.69	89 ± 5.07a
Azadirachtin-ELS	20 ± 4.71	30 ± 9.95	52 ± 12.29	62 ± 13.71	83 ± 1.18a
Azadirachtin-LS	17 ± 8.82	35 ± 4.19	47 ± 2.72	77 ± 7.93	97 ± 1.71a
**Statistical output**	*F*_11,36_ = 0.56; *P* = 0.85	*F*_11,36_ = 1.98; *P* = 0.06	*F*_11,36_ = 1.30; *P* = 0.26	*F*_11,36_ = 0.80; *P* = 0.64	*F*_11,36_ = 3.99; *P* = 0.001
**Conrad**					
Water-ES	10 ± 7.93	25 ± 5.69	33 ± 9.81	57 ± 12.32	84 ± 5.78
Water-ELS	17 ± 9.43	28 ± 9.95	45 ± 12.58	58 ± 6.31	79 ± 5.81
Water-LS	17 ± 8.82	27 ± 2.72	48 ± 8.33	53 ± 9.43	74 ± 4.63
Actigard-ES	10 ± 5.00	15 ± 4.19	45 ± 11.01	60 ± 4.71	70 ± 6.15
Actigard-ELS	13 ± 4.20	17 ± 4.30	30 ± 12.91	58 ± 5.69	60 ± 6.49
Actigard-LS	9 ± 8.50	13 ± 3.85	35 ± 6.31	50 ± 4.30	51 ± 4.30
*cis*-jasmone-ES	5 ± 5.00	15 ± 3.19	40 ± 9.81	57 ± 5.77	78 ± 4.63
*cis*-jasmone-ELS	17 ± 5.00	37 ± 6.94	33 ± 7.20	52 ± 8.33	57 ± 6.08
*cis*-jasmone-LS	10 ± 4.30	20 ± 4.71	55 ± 6.87	60 ± 13.61	71 ± 3.33
Azadirachtin-ES	15 ± 5.00	27 ± 8.16	38 ± 4.19	58 ± 13.44	65 ± 5.31
Azadirachtin-ELS	12 ± 5.00	15 ± 3.19	33 ± 6.09	53 ± 8.16	56 ± 9.97
Azadirachtin-LS	10 ± 4.30	23 ± 6.94	45 ± 15.24	68 ± 5.69	84 ± 6.61
**Statistical output**	*F*_11,36_ = 0.42; *P* = 0.93	*F*_11,36_ = 1.54; *P* = 0.16	*F*_11,36_ = 0.61; *P* = 0.80	*F*_11,36_ = 0.30; *P* = 0.98	*F*_11,36_ = 1.20; *P* = 0.52

**Notes:**

Mean values within columns (30 and 50 DAT at Knees and Choteau locations, respectively) bearing the different letters are significantly different (Tukey test, *P* < 0.05). The number of replicates per treatment was four.

PT, Pre-treatment; DAT, Days after the initial treatment application (i.e., chemical sprayed at wheat stem sawfly egg stage). ES, Egg stage; ELS, Egg and larval stages; LS, Larval stage.

Similarly, at the Conrad and Choteau locations, treatments had no significant effects on WSS infested damage percentage levels at any sampling time (Table 1). Except, at the final sampling time (i.e., 50 days after initial treatment application) at Choteau location, stem damage levels were found to be significantly lower on wheat plots treated with two times applications (WSS egg and larval stage) of Actigard^®^ (mean ± SE; 80% ± 1.50) compared with untreated control plots (92.00% ± 4.52 to 96.00% ± 1.65) (Table 1).

### Wheat stem sawfly and parasitoid population level

In general, WSS adult populations were found to be higher at the Choteau location followed by the Knees and Conrad locations (Table 2). The highest number of adults were observed at 10 and 20 days after initial treatment. Regardless of location, treatments did not have a significant impact on WSS adult populations, at any sampling time (Table 2).

**Table 2:** Effects of synthetic plant defense elicitors (Actigard^®^ & *cis*-jasmone) & Azadirachtin^®^ applications on wheat stem sawfly (WSS) & *Bracon* spp. adult individuals (15 sweeps/plot) & parasitism percentage (mean ± SE) in winter wheat fields at the three study locations of Montana.

Treatment	WSS adult number			Parasitoid adult number			Parasitism %
	10 DAT	20 DAT	30 DAT	10 DAT	20 DAT	30 DAT	
**Knees**							
Water-ES	7.50 ± 0.87	3.25 ± 1.31	0.5 0 ± 0.29	0.25 ± 0.25	3.25 ± 1.65	0.25 ± 0.25	24.05 ± 3.20
Water-ELS	5.50 ± 0.63	3.00 ± 0.91	0.00 ± 0.00	1.25 ± 0.41	2.75 ± 1.7	2.25 ± 0.95	14.83 ± 7.80
Water-LS	5.00 ± 1.22	1.75 ± 0.75	0.00 ± 0.00	0.25 ± 0.29	2.25 ± 0.48	1.25 ± 0.41	17.91 ± 9.00
Actigard-ES	2.25 ± 0.75	3.25 ± 0.25	0.25 ± 0.25	0.50 ± 0.25	4.50 ± 1.55	1.00 ± 0.41	13.48 ± 3.00
Actigard-ELS	1.15 ± 0.05	2.50 ± 0.87	0.00 ± 0.00	0.50 ± 0.00	4.00 ± 1.08	1.50 ± 0.65	9.32 ± 5.30
Actigard-LS	4.25 ± 1.03	1.75 ± 0.75	0.00 ± 0.00	0.00 ± 0.50	2.50 ± 1.5	1.50 ± 0.5	15.62 ± 3.10
*cis*-jasmone-ES	5.00 ± 1.68	2.00 ± 0.41	0.00 ± 0.00	0.25 ± 1.25	6.00 ± 2.35	0.00 ± 0.00	7.77 ± 1.90
*cis*-jasmone-ELS	3.75 ± 1.18	3.5 ± 1.19	0.00 ± 0.00	0.50 ± 0.25	4.75 ± 2.06	1.75 ± 0.63	14.72 ± 4.70
*cis*-jasmone-LS	2.75 ± 1.49	1.75 ± 0.85	0.50 ± 0.29	1.00 ± 0.75	4.25 ± 0.85	0.75 ± 0.48	13.21 ± 6.20
Azadirachtin-ES	4.25 ± 1.49	3.00 ± 0.41	0.25 ± 0.25	0.50 ± 0.25	4.00 ± 0.91	1.50 ± 0.5	19.84 ± 8.00
Azadirachtin-ELS	2.00 ± 0.40	3.25 ± 1.11	0.25 ± 0.25	1.25 ± 0.75	5.25 ± 1.31	1.50 ± 0.5	8.33 ± 8.30
Azadirachtin-LS	3.75 ± 1.93	2.75 ± 1.11	0.25 ± 0.25	0.25 ± 0.75	2.75 ± 1.11	0.75± 0.48	11.08 ± 4.00
**Statistical output**	${\rm{\chi }}_{11}^2\, = \,16.30$; *P* = 0.13	${\rm{\chi }}_{11}^2\, = \,7.38$; *P* = 0.77	${\rm{\chi }}_{11}^2\, = \,11.75$; *P* = 0.38	${\rm{\chi }}_{11}^2\, = \,16.64$; *P* = 0.47	${\rm{\chi }}_{11}^2\, = \,5.80$; *P* = 0.89	${\rm{\chi }}_{11}^2\, = \,13.38$; *P* = 0.27	${\rm{\chi }}_{11}^2\, = \,0.97$; *P* = 0.49
**Choteau**							
Water-ES	11.25 ± 1.89	19.00 ± 1.96	1.25 ± 0.63	2.00 ± 1.08	2.00 ± 0.41	0.25 ± 0.25	2.42 ± 1.02
Water-ELS	11.75 ± 2.06	19.75 ± 2.29	1.50 ± 0.87	2.25 ± 1.11	1.75 ± 1.11	2.75 ± 1.11	2.03 ± 1.43
Water-LS	12.25 ± 1.80	13.50 ± 1.19	1.00 ± 0.41	1.50 ± 0.75	2.50 ± 1.25	1.50 ± 0.76	1.56 ± 1.56
Actigard-ES	5.75 ± 1.38	21.50 ± 3.77	0.50 ± 0.50	1.75 ± 1.03	2.00 ± 0.82	2.00 ± 1.22	1.9 ± 0.68
Actigard-ELS	8.50 ± 1.44	15.25 ± 1.89	1.75 ± 0.85	0.50 ± 0.29	2.50 ± 1.19	0.75 ± 0.48	3.49 ± 1.35
Actigard-LS	10.50 ± 2.53	13.00 ± 3.00	1.75 ± 0.75	2.75 ± 1.18	2.75 ± 1.31	1.50 ± 1.19	2.9 ± 1.39
*cis*-jasmone-ES	11.00 ± 2.04	20.25 ± 3.47	2.25 ± 0.85	1.00 ± 0.71	1.50 ± 0.50	1.25 ± 0.75	2.27 ± 0.40
*cis*-jasmone-ELS	10.50 ± 2.10	17.75 ± 5.36	2.50 ± 1.19	1.25 ± 0.48	4.25 ± 0.85	0.75 ± 0.48	0.40 ± 0.40
*cis*-jasmone-LS	9.25 ± 2.21	18.25 ± 3.68	1.75 ± 0.63	1.50 ± 0.65	2.75 ± 0.95	0.75 ± 0.48	0.00 ± 0.00
Azadirachtin-ES	8.75 ± 3.47	20.00 ± 4.36	3.00 ± 0.71	1.75 ± 1.18	1.50 ± 0.65	1.50 ± 0.65	0.00 ± 0.00
Azadirachtin-ELS	6.25 ± 0.82	15.75 ± 3.82	0.50 ± 0.50	2.75 ± 1.6	3.50 ± 0.50	1.00 ± 0.58	0.78 ± 0.78
Azadirachtin-LS	6.75 ± 1.93	19.00 ± 3.16	1.00 ± 0.00	1.00 ± 0.71	1.25 ± 0.75	1.50 ± 0.96	0.00 ± 0.00
**Statistical output**	${\rm{\chi }}_{11}^2\, = \,14.67$; *P* = 0.20	${\rm{\chi }}_{11}^2\, = \,9.33$; *P* = 0.60	${\rm{\chi }}_{11}^2\, = \,12.74$; *P* = 0.31	${\rm{\chi }}_{11}^2\, = \,5.85$; *P* = 0.89	${\rm{\chi }}_{11}^2\, = \,12.32$; *P* = 0.34	${\rm{\chi }}_{11}^2\, = \,7.24$; *P* = 0.78	${\rm{\chi }}_{11}^2\, = \,1.23$; *P* = 0.30
**Conrad**							
Water-ES	3.00 ± 0.70	1.50 ± 0.95	0.50 ± 0.29	0.25 ± 0.25	0.25 ± 0.25	0.25 ± 0.25	10.54 ± 2.63
Water-ELS	2.50 ± 0.65	2.00 ± 1.08	0.00 ± 0.00	1.00 ± 0.41	0.75 ± 0.48	0.75 ± 0.48	7.39 ± 2.50
Water-LS	1.25 ± 0.48	1.00 ± 0.70	0.75 ± 0.48	1.00 ± 0.29	1.50 ± 0.29	0.00 ± 0.00	15.56 ± 3.04
Actigard-ES	2.67 ± 1.15	1.00 ± 0.70	0.25 ± 0.25	0.25 ±0.25	1.00 ± 0.71	0.25 ± 0.25	21.94 ± 3.94
Actigard-ELS	0.75 ± 0.48	0.75 ± 0.48	0.25 ± 0.25	0.00 ± 0.00	1.50 ± 0.87	0.5 ± 0.29	19.31± 6.97
Actigard-LS	1.75 ± 1.03	2.25 ± 1.31	0.00 ± 0.00	0.50 ±0.5	0.25 ± 0.25	0.00 ± 0.00	16.12 ± 1.98
*cis*-jasmone-ES	1.25 ± 0.63	1.00 ± 0.70	0.75 ± 0.48	1.25 ± 1.25	1.75 ± 0.85	0.0 0 ± 0.00	8.89 ± 5.11
*cis*-jasmone-ELS	2.50 ± 0.87	1.25 ± 0.75	0.25 ± 0.25	0.25 ± 0.25	1.25 ± 0.63	0.75 ± 0.25	10.92 ± 5.19
*cis*-jasmone-LS	2.50 ± 0.64	0.25 ± 0.25	0.00 ± 0.00	1.25 ± 0.75	1.50 ± 0.87	0.25 ± 0.25	18.08 ± 3.71
Azadirachtin-ES	1.00 ± 0.71	1.25 ± 0.75	0.25 ± 0.25	0.25 ± 0.25	1.00 ± 0.71	0.75 ± 0.75	7.67 ± 3.35
Azadirachtin-ELS	1.50 ± 0.87	0.75 ± 0.48	0.00 ± 0.00	1.25 ± 0.75	0.25 ± 0.25	0.25 ± 0.25	11.77 ± 6.06
Azadirachtin-LS	1.75 ± 0.85	0.75 ± 0.48	0.00 ± 0.00	0.75 ± 0.75	0.25 ± 0.25	0.25 ± 0.25	12.38 ± 6.79
**Statistical output**	${\rm{\chi }}_{11}^2\, = \,10.42$; *P* = 0.49	${\rm{\chi }}_{11}^2\, = \,3.66$; *P* = 0.98	${\rm{\chi }}_{11}^2\, = \,10.41$; *P* = 0.51	${\rm{\chi }}_{11}^2\, = \,13.70$; *P* = 0.25	${\rm{\chi }}_{11}^2\, = \,8.33$; *P* = 0.68	${\rm{\chi }}_{11}^2\, = \,16.30$10.65; *P* = 0.47	${\rm{\chi }}_{11}^2\, = \,16.30$1.05; *P* = 0.43

**Notes:**

The number of replicates per treatment was four.

DAT, Days after the initial treatment application (i.e., chemical sprayed at wheat stem sawfly egg stage); ES, Egg stage; ELS, Egg and larval stages; LS, Larval stage.

Higher *Bracon* spp. adult population levels were observed at the Knees location followed by Choteau and Conrad locations (Table 2). There were no significant differences in parasitoid adult population levels between treatments at each sampling time at the Knees, Choteau, and Conrad locations (Table 2). In addition, the study did not deduce any significant difference on parasitism levels between treatments (Table 2). The overall average parasitism at the Knees, Choteau, and Conrad locations varied from 7.77% to 25.05%, 0.00–3.49%, and 7.67–21.94%, respectively (Table 2).

### Diapausing larval body weight

**Figure 4:**
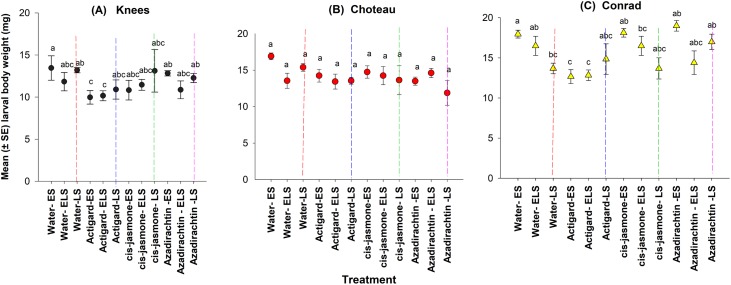
Effects of synthetic plant defense elicitors (Actigard^®^ and *cis*-jasmone) and Azadirachtin^®^ applications on the body weight of diapausing larvae (mean ± SE) at the three study locations of Montana. ES, Egg stage; ELS, Egg and larval stages; LS, Larval stage. (A) Knees, (B) Choteau, (C) Conrad. Circle or triangle of each color bearing the different letters are significantly different (Tukey test, *P* < 0.05). The number of replicates per treatment was four.

Overall, diapausing WSS larvae body weights were observed higher at the Conrad location, followed by Choteau and Knees locations (Fig. 4). Mean levels of body weight in chemical-treated or untreated control plots ranged from 12.66 to 19.00 mg for Conrad, 11.88–16.90 mg for Choteau and 9.67–13.46 mg for Knees locations (Fig. 4). Overall, chemical type (*F* = 9.13; d*f* = 3, 135; *P* < 0.0001) and timing of treatment application (*F* = 3.78; d*f* = 2, 135; *P* = 0.02) had significant effects on diapausing larval body weight, based on the linear mixed model analyses. No significant interaction (*F* = 1.91; d*f* = 6, 129; *P* = 0.80) between the chemical type and timing of treatment application was detected and consequently, the parameter estimates from the additive mixed model (chemical + application timing) were presented (Table 3).

**Table 3:** Linear mixed model fitted to wheat stem sawfly larval body weight (mg), larval mortality (%) and wheat stem lodging (1-10 rating) data with main effects for treatment (chemical type) and application timing and a location for random effect.

Fixed effect	Parameter estimate	SE	*t* value	*P*
**Body weight**				
(Intercept)	12.90	1.12	11.48 (135)	<0.001
Treatment (*cis*-jasmone)	1.86	0.53	3.49 (135)	0.01
Treatment (Control)	2.69	0.53	5.05 (135)	<0.0001
Treatment (Azadirachtin^®^)	1.84	0.54	3.44 (135)	<0.001
Application timing (egg and larva stage)	−1.22	0.46	−2.66 (135)	0.01
Application timing (larval stage)	−0.89	0.46	−1.99 (135)	0.06
**Larval mortality**				
(Intercept)	33.33	4.81	6.93 (130)	<0.001
Treatment (*cis*-jasmone)	−9.00	5.42	−1.66 (130)	0.10
Treatment (Control)	−15.25	5.42	−2.81 (130)	0.01
Treatment (Azadirachtin^®^)	−7.25	5.42	−1.33 (130)	0.18
Application timing (egg and larva stage)	11.25	5.42	2.08 (130)	0.04
Application timing (larval stage)	−5.67	5.42	−1.05 (130)	0.29
*cis*-jasmone*egg and larva stage	−8.17	7.67	−1.14 (130)	0.26
Control*egg and larval stage	−8.50	7.67	−1.11 (130)	0.27
Azadirachtin^®^ *egg and larval stage	−26.50	7.67	−3.46 (130)	<0.001
*cis*-jasmone*larval stage	13.83	7.67	1.80 (130)	0.07
Control*larval stage	−6.58	7.67	0.86 (130)	0.39
Azadirachtin^®^ *larval stage	−6.58	7.67	1.47 (130)	0.14
**Stem lodging**				
(Intercept)	5.36	0.70	7.92 (138)	<0.001
Treatment (*cis*-jasmone)	1.88	0.34	5.54 (138)	<0.001
Treatment (Control)	2.69	0.34	7.91 (138)	<0.001
Treatment (Azadirachtin^®^)	1.11	0.34	3.26 (138)	0.001

**Note:**

Parameter estimates are with reference to Actigard^®^ treatment and application timing at egg stage. Degrees of freedom are given in parenthesis.

This study demonstrated main significant effect on larval body weight for both chemical type and timing of treatment application at two of the study locations: Knees (chemical type: *F* = 6.38; d*f* = 3, 44; *P* = 0.001; timing of treatment application: *F* = 3.35; d*f* = 11, 36; *P* = 0.002) and Conrad (chemical type: *F* = 4.43; d*f* = 3, 44; *P* = 0.008; timing of treatment application: *F* = 4.89; d*f* = 11, 36; *P* = 0.0001) (Fig. 4). There was a significantly lower larval body weights on Actigard-treated plots with one application (WSS egg stage) and two times applications (WSS egg and larval stages) compared to untreated control plots at the Knees location (Fig. 4). At the Conrad location, lower body weight was significantly observed with Actigard^®^ two applications made at WSS egg and larval stages compared to most of the untreated control plots (Fig. 4). In contrast, there was no significant main effect on larval body weight for chemical type (*F* = 2.22; d*f* = 3, 44; *P* = 0.09) and timing of treatment application (*F* = 1.85; d*f* = 11, 36; *P* = 0.08) at the Choteau location. There were significant interactions between chemical type and timing of treatment application at the Conrad location (*F* = 4.10; d*f* = 8, 44; *P* = 0.001), but not at the Knees (*F* = 1.84; d*f* = 8, 44; *P* = 0.10) or Choteau (*F* = 1.62; d*f* = 8, 44; *P* = 0.15). Regardless of treatment application timing, *cis*-jasmone and Azadirachtin^®^ treatments had no effect on larval body weight at the Knees and Choteau locations (Fig. 4).

### Diapausing larval mortality

Overall, linear mixed model analyses indicated that chemical type (*F* = 6.31; d*f* = 9, 130; *P* = 0.0001) and application timing (*F* = 3.57; d*f* = 8, 130; *P* = 0.0001) had significant effects on diapausing larval mortality. Significant interaction (*F* = 4.63; d*f* = 6, 130; *P* < 0.0001) was found between the chemical type and application timing and the parameter estimates from interaction model (chemical type × application timing) are presented (Table 3).

**Figure 5:**
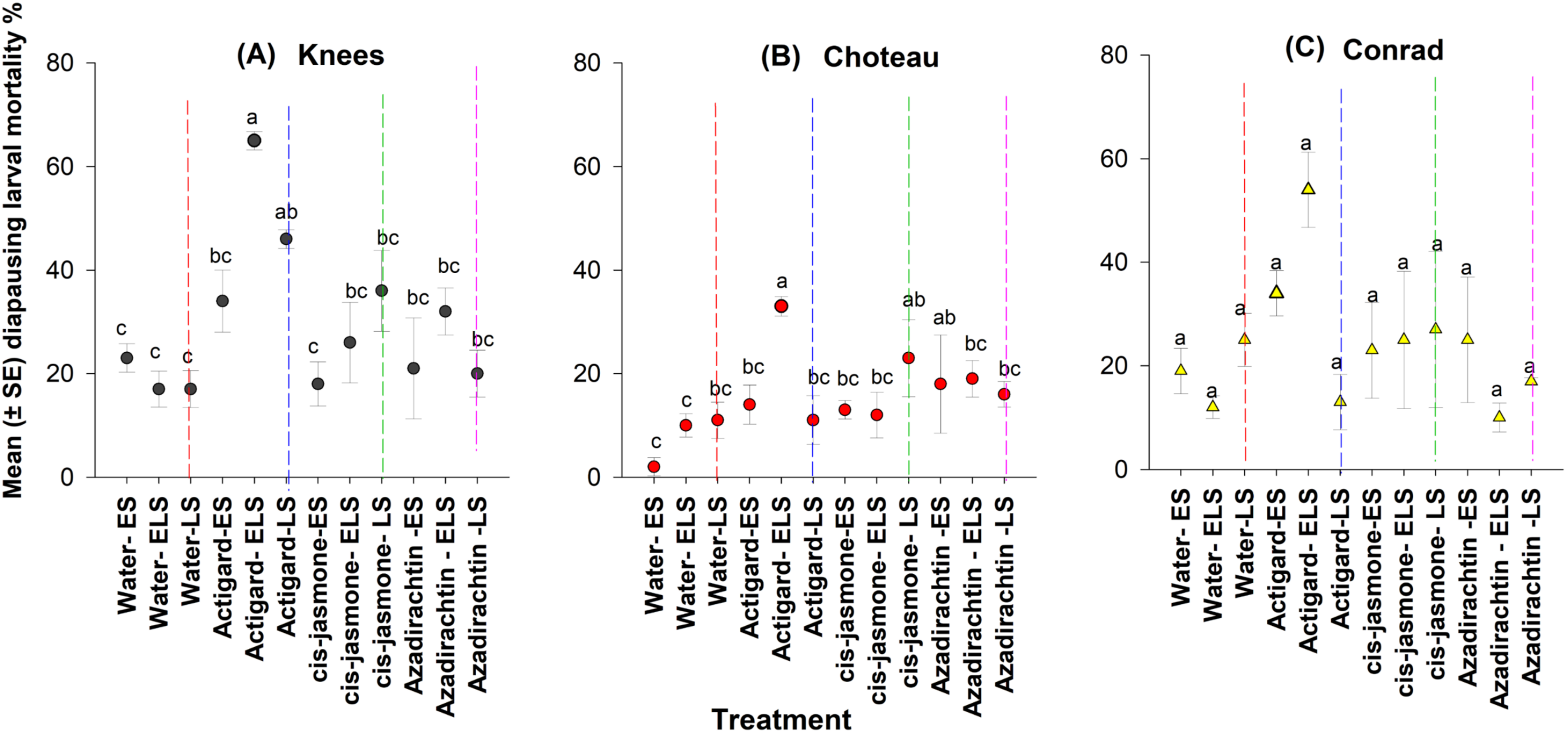
Effects of synthetic plant defense elicitors (Actigard^®^ and *cis*-jasmone) and Azadirachtin^®^ applications on total mortality of diapausing larvae (mean ± SE), recorded in dissected stems at final harvest in winter wheat at the three study locations of Montana. ES, Egg stage; ELS, Egg and larval stages; LS, Larval stage. (A) Knees, (B) Choteau, (C) Conrad. Circle or triangle of each color bearing the different letters are significantly different (Tukey test, *P* < 0.05). The number of replicates per treatment was four.

This study depicted significant main effect on diapausing larval mortality for both chemical type (Knees: *F* = 9.32; d*f* = 3, 44; *P* = 0.0001 and Choteau: *F* = 3.40; d*f* = 3, 44; *P* = 0.02) and application timing (Knees: *F* = 4.78; d*f* = 11, 36; *P* = 0.0001 and Choteau: *F* = 4.79; d*f* = 11, 36; *P* = 0.0001) (Fig. 5). There were significant chemical-application timing interactions at both Knees (*F* = 2.27; d*f* = 8, 44; *P* = 0.04) and Choteau (*F* = 4.50; d*f* = 8, 44; *P* = 0.001) locations.

At the Knees location, across the timing of treatment application, significantly higher diapausing larval mortality occurred when Actigard^®^ was applied two times (WSS egg and larval stages) compared to untreated control wheat plots (Fig. 5). Delayed one-time application (WSS larval stage) of Actigard^®^ had significantly higher larval mortality than untreated control plots, but its initial application (WSS egg stage) had no effect on mortality (Fig. 5). Similarly, at the Choteau location, wheat plots treated twice (WSS egg and larval stages) with Actigard^®^ had significantly higher larval mortality when compared to untreated control plots. In addition, delayed one-time application of *cis*-jasmone and Azadirachtin^®^ had significantly higher larval mortality compared to untreated control plots sprayed with water at the WSS egg stage (Fig. 5).

However, in Conrad location, chemical type had the only significant main effect on larval mortality (*F* = 3.18; d*f* = 11, 44; *P* = 0.03), whereas timing of treatment application was without effect on larval mortality (*F* = 1.96; d*f* = 11, 36; *P* = 0.06) (Fig. 5). There were no significant interactions between chemical type and timing of treatment application (*F* = 1.41; d*f* = 8, 44; *P* = 0.22).

### Wheat stem lodging at harvest

Based on overall data fitted to the linear mixed model, chemical type (*F* = 23.15; d*f* = 3, 136; *P* = 0.0001) had significant impact on wheat stem lodging, but without any effect of timing of treatment application (*F* = 2.37; d*f* = 2, 136; *P* = 0.09). No significant interaction (*F* = 1.91; d*f* = 6, 130; *P* < 0.08) was found between the chemical type and timing of treatment application and the parameter estimates from reduced mixed model (chemical type) are presented (Table 3).

**Figure 6:**
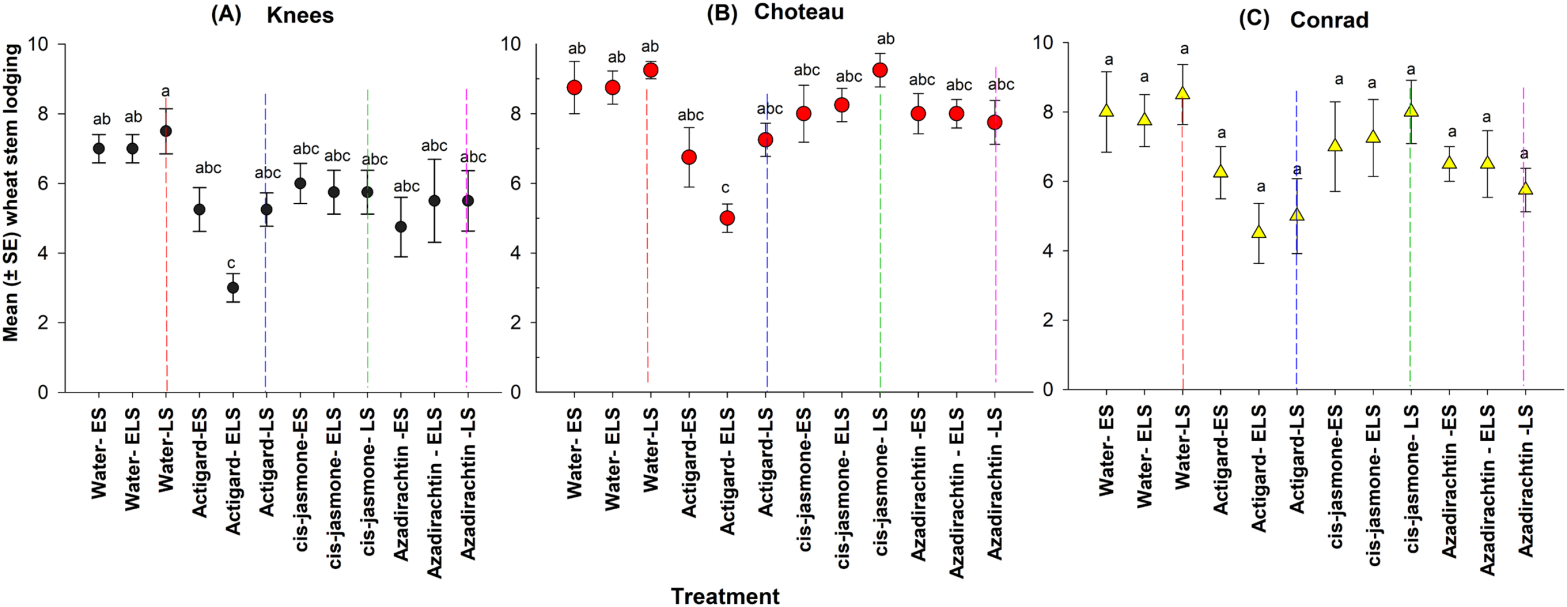
Effects of synthetic plant defense elicitors (Actigard^®^ and *cis*-jasmone) and Azadirachtin^®^ applications on the wheat stem lodging (mean ± SE) recorded at the harvesting time at the three study locations of Montana. In *y*-axis scale, 1 indicates that all plants in a plot were vertical and 10 indicates that all plants in a plot were horizontal during crop harvest time. ES, Egg stage; ELS, Egg and larval stages; LS, Larval stage. (A) Knees, (B) Choteau, (C) Conrad. Circle or triangle of each color bearing the different letters are significantly different (Tukey test, *P* < 0.05). The number of replicates per treatment was four.

This study demonstrated main significant effect on wheat stem lodging for both chemical type and timing of treatment application at two of the study locations: Knees (chemical type: *F* = 8.12; d*f* = 3, 44; *P* = 0.0001; timing of treatment application *F* = 3.02; d*f* = 11, 36; *P* = 0.001) and Choteau (chemical type: *F* = 10.66; d*f* = 3, 44; *P* = 0.0001; timing of treatment application *F* = 4.20; d*f* = 11, 36; *P* = 0.0001) (Fig. 6). At both locations, significantly lower mean stem lodging (±SE) occurred when Actigard^®^ was applied twice (WSS egg and larval stages) (Knees: 3.00 ± 0.40; Choteau: 5.00 ± 0.41) while the remaining treatments showed no significant differences from the untreated controls (Knees: 7.00–7.50; Choteau: 8.75–9.00) (Fig. 6). There were no interaction effects between treatment and timing of treatment application (Knees: *F* = 1.06; d*f* = 8, 44; *P* = 0.41 and Choteau: *F* = 1.45; d*f* = 8, 44; *P* = 0.20).

At the Conrad location, treatment was the only main factor that significantly affected the wheat stem lodging rate (*F* = 6.09; d*f* = 3, 44; *P* = 0.01), but without significant effect for timing of treatment application (*F* = 1.78; d*f* = 11, 36; *P* = 0.09) (Fig. 6) and interaction (*F* = 0.41; d*f* = 8, 44; *P* = 0.90).

### Yield

Irrespective of treatment and location, wheat plots treated with *cis*-jasmone had higher yields than plots treated with Actigard^®^ and Azadirachtin^®^ (Table 4). However, there were no significant differences on yield levels between treatments at any location (Knees: *F* = 0.635; d*f* = 11, 36; *P* = 0.78; Choteau: *F* = 0.50; d*f* = 11, 36; *P* = 0.89; and Conrad: *F* = 0.28; d*f* = 11, 36; *P* = 0.98). Average winter wheat grain yield levels ranged from 3,660–4,077, 3,613–3,972, and 4,349–5,241 kg/ha at the Knees, Choteau and Conrad locations, respectively (Table 4).

**Table 4:** Effects of synthetic plant defense elicitors (Actigard^®^ and *cis*-jasmone) and Azadirachtin^®^ applications on average yield and quality (± SE) parameters of winter wheat fields infested at the three study locations of Montana.

Treatment	Yield (kg/ha)	Test weight (kg/m^3^)	Protein (%)
**Knees**			
Water-ES	3900 ± 186.90	788 ± 1.43	11 ± 0.05
Water-ELS	3966 ± 176.98	787 ± 4.44	11 ± 0.27
Water-LS	3729 ± 127.53	790 ± 6.15	12 ± 0.36
Actigard-ES	3660 ± 246.29	795 ± 1.22	11 ± 0.18
Actigard-ELS	3686 ± 198.49	797 ± 2.61	12 ± 0.18
Actigard-LS	3809 ± 224.53	795 ± 1.39	12 ± 0.23
*cis*-jasmone-ES	4045 ± 150.30	790 ± 2.81	11 ± 0.33
*cis*-jasmone-ELS	4077 ± 89.42	793 ± 3.66	11 ± 0.13
*cis*-jasmone-LS	3823 ± 144.05	792 ± 0.37	11 ± 0.45
Azadirachtin-ES	4027 ± 161.50	792 ± 1.75	11 ± 0.19
Azadirachtin-ELS	3807 ± 224.22	794 ± 0.91	11 ± 0.21
Azadirachtin-LS	3932 ± 142.87	791 ± 1.85	11 ± 0.21
***P value***	NS	NS	NS
**Choteau**			
Water-ES	3798 ± 311.60	770 ± 8.53	14 ± 0.29
Water-ELS	3810 ± 184.51	771 ± 7.65	13 ± 0.40
Water-LS	3763 ± 154.44	768 ± 8.27	14 ± 0.40
Actigard-ES	3893 ± 423.80	766 ± 5.27	15 ± 0.40
Actigard-ELS	3923 ± 373.88	789 ± 1.22	14 ± 0.30
Actigard-LS	3972 ± 261.37	776 ± 1.62	15 ± 0.18
*cis*-jasmone-ES	3949 ± 382.61	764 ± 7.60	13 ± 1.03
*cis*-jasmone-ELS	3719 ± 134.89	777 ± 5.27	13 ± 0.42
*cis*-jasmone-LS	3686 ± 183.94	767 ± 7.71	15 ± 0.13
Azadirachtin-ES	3677 ± 82.25	765 ± 9.79	14 ± 0.53
Azadirachtin-ELS	3613 ± 129.84	755 ± 8.14	14 ± 0.31
Azadirachtin-LS	3894 ± 166.76	768 ± 7.32	14 ± 0.45
***P-value***	NS	NS	NS
**Conrad**			
Water-ES	4393 ± 817.03	793 ± 3.83	13 ± 0.32
Water-ELS	4827 ± 528.68	798 ± 6.41	13 ± 0.53
Water-LS	4679 ± 480.06	789 ± 2.50	13 ± 0.23
Actigard-ES	4730 ± 587.90	796 ± 4.68	13 ± 0.32
Actigard-ELS	4652 ± 402.65	791 ± 6.85	13 ± 0.28
Actigard-LS	5003 ± 725.85	793 ± 6.60	13 ± 0.39
*cis*-jasmone-ES	4828 ± 535.94	819 ± 14.03	14 ± 0.95
*cis*-jasmone-ELS	5241 ± 340.76	796 ± 5.50	13 ± 0.20
*cis*-jasmone-LS	4600 ± 317.66	790 ± 4.99	13 ± 0.23
Azadirachtin-ES	5465 ± 654.48	796 ± 4.02	13 ± 0.39
Azadirachtin-ELS	5128 ± 574.57	794 ± 5.49	13 ± 0.47
Azadirachtin-LS	5203 ± 751.90	788 ± 7.15	13 ± 0.44
***P-value***	NS	NS	NS

**Note**

The number of replicates per treatment was four.

ES, Egg stage; ELS, Egg and larval stages; LS, Larval stage; NS, no significant.

### Quality

Treatments had no significant impact on kernel weight at any location: Knees (*F* = 1.67; d*f* = 11, 36; *P* = 0.12), Choteau (*F* = 1.09; d*f* = 11, 36; *P* = 0.40) and Conrad (*F* = 1.53; d*f* = 11, 36; *P* = 0.16) (Table 4). Similarly, there were no significant differences in protein percentage between treatment at any study location: Knees (*F* = 0.70; d*f* = 11, 36; *P* = 0.73), Choteau (*F* = 1.49; d*f* = 11, 36; *P* = 0.17) and Conrad (*F* = 0.73; d*f* = 11, 36; *P* = 0.69). The test weight across all treatment ranged from 788–819 kg/m^3^ for Conrad, 790–795 kg/m^3^ for Knees, and 755–789 kg/m^3^ for Choteau locations (Table 4). Protein levels across all treatment varied from 13–15% for Choteau, 13–14% for Conrad and 11–12% for Knees locations (Table 4).

## Discussion

Caged adult behavior assays showed that Actigard^®^ and Azadirachtin^®^ treatments influenced WSS adults preference behavior, with a significantly fewer adults observed on treated plants than on untreated control plants. No previous information exists regarding WSS adults preference between Actigard^®^, *cis*-jasmone or Azadirachtin^®^ treated plants over untreated control plants. However, our results were in accordance with previous studies, indicating that Actigard^®^ (Inbar et al., 1998; Moran & Thompson, 2001; Cooper, Jia & Goggin, 2004; Correa et al., 2005) and Azadirachtin^®^ (Hunter & Ullman, 1992; Lowery & Isman, 1993; Musabyimana et al., 2001) may have abilities to repel insect pests. For examples, Moran & Thompson (2001) and Cooper, Jia & Goggin (2004) showed that tomato seedlings treated with Actigard^®^ was deterrent to both leaf miner *Liriomyza trifolii* (Burgess) and potato aphid *Macrosiphum euphorbiae* (Thomas) compared to untreated control plants. Similarly, Lowery & Isman (1993) and Musabyimana et al. (2001) demonstrated that strawberry leaves and banana corms treated with Azadirachtin^®^ repelled the strawberry aphid, *Chaetosiphon fragaefolii* (Cockerell) and the banana root borer, *Cosmopolites sordidus* (Germar), respectively.

In contrast, in field situations, regardless of sampling time and field location, this study did not find significant differences in WSS adult populations between treated wheat plots and untreated controls. Specifically, at the 10 days after initial treatment application in all field locations, there was nearly threefold less WSS adult populations on wheat plots treated with Actigard^®^ and Azadirachtin^®^ at WSS egg and larval stages (i.e., spraying twice) compared with untreated control plots. The lack of significant treatment effects in our study was likely due to few replications which resulted in a lot of variation in mean adult captures. Nevertheless, based on lab and field observations, it is expected that Actigard^®^ and Azadirachtin^®^ applied at both egg and larval stages may have some ability to repel the WSS adults from ovipositing into stems in fields, but it also warrants additional studies to validate these observations.

The field study demonstrated that Actigard^®^ treatments negatively affected WSS body weight and survival. Larvae in treated plots had reduced body weight and higher mortality compared with untreated control plots. This study further indicated that repeated application (i.e., two sprayings) may be necessary to enhance mortality, as Actigard^®^ applied at WSS egg and larval stages inflicted significantly higher larval mortality than untreated controls at the Knees and Choteau location, but with no effect at the Conrad location. In addition, larval body weight was found to be significantly lower for plots treated with Actigard^®^ at WSS egg stage (applied once), and egg and larval (applied twice) stages in contrasted to untreated control plots at the Knees and Conrad locations, but without effects at the Choteau location.

Our results agree with other studies that have observed a negative effect on insect fitness when they were fed Actigard-treated plants. Actigard^®^ applications reduced reproduction and body weight for whiteflies, *Bemisia tabaci* (Gennadius) fed on treated cucumber (Correa et al., 2005), and increased mortality of soybean loopers, *Chrysodeixis includens* (Walker) fed on treated soybean plants (Chen et al., 2018). Similarly, decreases in larval and pupal body weights of sunflower caterpillar, *Chlosyne lacinia saundersii* (Doubleday), and second instar nymphs of *Bemisia tabaci* have been reported when these insects fed on Actigard^®^ treated sunflower (Assis et al., 2015) and tomato (Nombela et al., 2009) plants, respectively.

However, the effects of Actigard^®^ treatments on insect fitness also appear to be influenced by the insect and plant species. For instances, Bi, Murphy & Felton (1997) and Inbar et al. (2001) found that Actigard-treated cotton plants had no effect on the fitness (e.g., mortality, growth, and development) of corn earworm, *Helicoverpa zea* (Boddie) and cotton bollworm, *H. armigera* (Hubner). In addition, crop variety can also influence the Actigard^®^ treatments effect on insects. Cooper, Jia & Goggin (2004) found lower population growth of potato aphids fed on Actigard-treated susceptible tomato cultivar but no such effect on aphids feeding on a resistant cultivar.

Plants are generally known to exhibit systemic resistance after exogenous application of Actigard. At least six defensive chemical compounds are known to be induced and present at higher levels in plants after Actigard^®^ application: (1) pathogenesis-related proteins, (2) chitinases, (3) β-1,3-glucanases, (4) phenolic acid, (5) peroxidase, and (6) lipoxygenase (Thaler et al., 1999; Inbar et al., 2001; Stout, Zehnder & Baur, 2002; Bektas & Eulgem, 2015; Ramos et al., 2017). The induction of any of these chemical compounds in Actigard-treated winter wheat plants could have been responsible for the observed reduction in WSS larval fitness in wheat after Actigard^®^ application. Specific to winter wheat plants, a recent study showed that Actigard^®^ exogenous application at wheat plants jointing stages enhanced total phenolic content in wheat bran extract when compared with untreated control plants (Ramos et al., 2017).

In contrast, Azadirachtin^®^ application to winter wheat plots did not significantly reduce WSS fitness (larval body weight and mortality) compared with untreated controls at any of the three field locations. However, the effect of Azadirachtin^®^ treatments on insect fitness can be influenced by insect taxonomic relationships. Azadirachtin^®^ usually has greater effects on members of the orders, Lepidoptera (Mordue & Nisbet, 2000; Montes-Molina et al., 2008) and Diptera (Hossain & Poehling, 2006; Bezzar-Bendjazia, Kilani-Morakchi & Aribi, 2016) than Hymenoptera (Mordue & Nisbet, 2000) and Coleoptera (Cherry & Nuessly, 2010). This suggests that Azadirachtin^®^ may not affect WSS larval to a great degree. In addition, Azadirachtin^®^ lower persistence or stability in field situations could have contributed further for such no effect, since previous studies have shown that Azadirachtin^®^ half-life can be less than 1 day in outdoor environments (Caboni et al., 2002).

With respect to *cis*-jasmone treatment, specifically at the Knees and Choteau locations, larval mortality was nearly twofold higher on winter wheat plots treated with this chemical at WSS larval stage inside stems compared to untreated control, although this difference was only significant at Choteau location. There are no obvious explanations for this observation and it remained unclear why two applications (WSS egg and larval stages) of *cis*-jasmone had no impact on larval mortality. However, as reported in the previous studies on other crop-pest systems (Thaler et al., 1999; Omer et al., 2000; Cooper, Jia & Goggin, 2004), it is possible that *cis*-jasmone applications during the larval stage may have induced winter wheat plants to produce toxic secondary metabolites that caused higher mortality in WSS larvae.

Our study also demonstrated that plots treated Actigard^®^ two times applications had significantly lower WSS infestation levels 30 and 50 days after initial treatment, at the Knees and Choteau, respectively. It is unclear why Actigard^®^ two times applications had a significant effect at 30 days, but not at 50 days at the Knees, this was an unexpected result. Since WSS adult emergence can last for about 1 month (Knodel, Shanower & Beauzay, 2016; Fulbright et al., 2017), late emergence might have influenced WSS adults flying and oviposition activity, and thereby later infestation levels in winter wheat fields. However, significantly lower stem lodging was found in plots treated with Actigard^®^ two times treatment application at both at the Knees and Choteau. This suggests that fewer WSS larvae were present in Actigard^®^ treated plots. We found no differences in WSS infestation levels or stem lodging in Azadirachtin^®^ or *cis*-jasmone treated plots. Tangtrakulwanich et al. (2014) reported that Azadirachtin^®^ application caused a significant reduction in WSS infestation levels in spring wheat when compared to untreated controls, 2–4 weeks after treatment application, but this report contrasts with our findings in this study. This may likely due to different crop growing seasons, environmental factors (e.g., temperatures and sunlight) or WSS population levels.

For other parameters including yield and quality levels, we did not find any significant differences in plots treated with chemicals, including Actigard^®^. The reason for this lack of effect on yield is unclear, but Actigard^®^ application has previously been found to reduce (Romero, Kousik & Ritchie, 2001) or have no impact on crop yields (Louws et al., 2001). Although our results did not show direct treatment effects on numbers of *Bracon* spp. adults and parasitism levels, on average, *cis*-jasmone treated plots had greater numbers of parasitoids and higher parasitism levels compared to other treatments. This agrees with previous studies indicating that *cis*-jasmone application enhances natural enemy populations (Moraes et al., 2009; Sobhy, Erb & Turlings, 2015).

## Conclusion

In conclusion, this preliminary research has provided information that may improve the utilization of synthetic plant defense elicitors for integrated pest management of WSS. The Actigard^®^ used in our study, particularly when applied twice (egg and larval stage inside stems), showed higher WSS larval mortality, reduced larval body weight, and less wheat stem lodging. On the other hand, Actigard^®^ applied twice did not reduce the numbers of WSS adults captured in the plots or improve wheat yield and quality. Additional field studies are necessary to determine whether Actigard^®^ application techniques (e.g., timing, rate, insect stage, and crop stage) can reduce WSS damage and improve wheat yield and quality. In addition, cost/benefit analysis has to determine, whether or not, Actigard^®^ applications are economical and sustainable for winter wheat producers. Overall, management of WSS using synthetic plant defense elicitors could be a potential option for managing WSS, especially in the absence of effective synthetic insecticides, and could be easily incorporated into an integrated pest management program.

## Supplemental Information

10.7717/peerj.5892/supp-1 Supplemental Information 1 Raw data.

## Additional Information and Declarations

### Data Availability

The following information was supplied regarding data availability: The raw data are provided in a Supplemental File.
